# Editorial: Podocyte Pathology and Nephropathy—An Update

**DOI:** 10.3389/fendo.2019.00528

**Published:** 2019-07-30

**Authors:** Sanna Lehtonen, Barbara Lewko

**Affiliations:** ^1^Department of Pathology and Research Program for Clinical and Molecular Metabolism, Faculty of Medicine, University of Helsinki, Helsinki, Finland; ^2^Department of Pathophysiology, Faculty of Pharmacy, Medical University of Gdansk, Gdańsk, Poland

**Keywords:** podocytes, diabetes, nephropathy, gut-kidney, GLUT, angiotensin II, nephrin, imaging

Dysfunction and loss of glomerular podocytes have been found to be the driving forces leading to the development of the majority of the forms of chronic kidney disease (CKD) (1, 2). Podocytes are unique cells not only in their complex form and function, but also due to their inability to proliferate and replenish the lost podocytes in the mature kidney. Since podocyte loss is irreversible, development of podocyte-protective therapies is of particular importance. The previous Research Topic on Podocyte Pathology and Nephropathy (3) covered diagnostic approaches and novel therapeutic strategies for early detection of and protection from podocyte injury. In the current issue, the authors present up-to-date findings on the potential targets for podocyte-specific drugs as well as on mechanisms underlying podocyte and kidney impairment. Particular emphasis is given to molecular mechanisms that may contribute to the pathogenesis of diabetic podocytopathy.

Diabetes is the most common cause of CKD and end-stage renal disease worldwide. Multiple pathways triggered by hyperglycemia in different tissues have also been observed to be active in podocytes. Podocyte injury is pivotal for the loss of renal function and, in line with this, podocytes are considered to be one of the weakest players in the kidney homeostasis disturbed in diabetic kidney disease (DKD) (4). Nevertheless, our knowledge on the pathophysiological mechanisms leading to podocyte injury and development of DKD is still incomplete. A recent study shed new light on this by sub-categorizing the patients with diabetes to distinct groups based on six clinical variables, revealing that severely insulin resistant patients with diabetes are at high risk to develop DKD (5). As highlighted by Lay and Coward, insulin modulates several physiological responses in podocytes, and insulin resistance has multiple consequences that contribute to podocyte impairment and the development of DKD. In line with this, podocyte-specific deletion of insulin receptor leads to the development kidney injury resembling DKD even though the mice remain normoglycemic. The pathomechanisms of insulin resistance in podocytes include defective action of nephrin, a protein expressed in the slit diaphragm and essential for the function of the glomerular filtration barrier. Nephrin can directly interact with the insulin receptor, and thereby may regulate its function and impact on insulin-dependent effects in podocytes. The authors also discuss the role of epigenetic modifications in the control of podocyte insulin signaling, and highlight the links between insulin signaling, endoplasmic reticulum stress, and mitochondrial function in DKD.

The intracellular level of glucose in podocytes is regulated by facilitative glucose transporters (GLUTs) similarly as in other insulin responsive cells. However, as indicated by Wasik and Lehtonen, the roles of GLUT proteins in the development of DKD seem to vary depending on the GLUT and the cell type in which it is expressed. Whereas, overexpression of GLUT1 and knockout of GLUT4 specifically in podocytes have been shown to protect against the development of DKD, high GLUT1 expression in mesangial cells is associated with the progression of DKD. Efficacy of glucose uptake by GLUTs is tightly correlated with the activity of the pathways that regulate the trafficking of GLUTs between the intracellular stores and the plasma membrane. Notably, nephrin participates in the control of the intracellular trafficking of GLUTs via interactions with the regulatory proteins. The present study by Lewko et al. further shows that the surface expression of GLUT1, GLUT2, and GLUT4 correlates with glucose uptake in podocytes and is directly regulated by angiotensin II (AngII). GLUT1- and GLUT4- mediated glucose transport depends on insulin-triggered signaling which is interfered by AngII. In hyperglycemic milieu glucose uptake in podocytes becomes insensitive to insulin but not to AngII.

Initially considered to be mainly a structural protein of the filtration barrier, nephrin appears to play far more complex roles in podocytes. In addition to mediating insulin-induced actions in podocytes as described above, nephrin, via phosphorylation of its various tyrosine and threonine residues, forms complexes with many proteins, as pointed out by Martin and Jones. Signaling networks established this way enable dynamic control of various cellular processes, including, for example, modulation of the actin cytoskeleton dynamics and mechanosensing, and thereby regulation of podocyte morphology and glomerular barrier function. Phosphorylation of nephrin regulates its endocytosis, and interestingly, nephrin trafficking both from the endoplasmic reticulum to the plasma membrane as well as from the plasma membrane back to the intracellular compartment are essential for the function of nephrin. The authors of the article thus propose that aberrant trafficking of nephrin may be a central mechanism of nephrin-associated kidney diseases.

Interorgan crosstalk is considered exceedingly important in the development of various diseases, including CKD. In their article Lehto and Groop describe gut-kidney interorgan crosstalk linking the gastrointestinal tract and the pathogenesis of nephropathy and associated podocyte injury. Gut with its microbiota forms the largest interface between the individual and the outside world. Not only nutrition, but also whole-body immunity depends on the condition of the gastrointestinal tract. It is now clear that disruption of the gastrointestinal wall and gut dysbiosis, or altered gut bacterial composition, are associated with translocation and accumulation of nephrotoxic compounds. Dysregulation of the whole-body immune system by alterations of the gut microbiota increases the risk of certain types of kidney disease. Based on putative interconnections between gastrointestinal and renal disorders, renoprotective effects of dietary interventions including probiotic and resistant starch supplementation as well as transplantation of fecal microbiota are discussed. Also potential novel therapeutic targets are introduced. In addition to the gut-kidney axis, interactions between adipose tissue and kidney (6) as well as between liver and kidney (7) play a role in normal kidney function and in response to kidney injury.

Studying podocyte biology using cells cultured in the two-dimensional system has inherent limitations. As highlighted by Lal and Patrakka, the ideal research model should recapitulate the basement membrane that is sandwiched between the layers of podocytes and endothelial cells. Thus, direct monitoring of podocytes *in vivo* would be the optimal tool for analyzing the morphology and behavior of these highly specialized cells. So far, due to optical resolution limits, electron microscopy has been the tool of choice to study podocyte foot processes and their altered morphology in disease states. However, recent years have brought about the development of different super-resolution microscopic techniques enabling significant new insights into podocyte morphology. Novel microscopy approaches and improved methods for sample preparation have allowed visualization of the morphological details of the podocytes and observation of podocyte behavior *in situ*. In their review, Siegerist et al. present current options for podocyte research using the latest microscopic techniques, including three-dimensional structured illumination microscopy (3D-SIM), stimulated emission depletion microscopy (STED), stochastic optical reconstruction microscopy (STORM), and photoactivated localization microscopy (PALM). In addition, multiphoton microscopy (MPM) and light-sheet/selective plane illumination microscopy (SPIN), allowing intravital imaging of podocytes, are described.

The title of the review by Lal and Patrakka, “*Understanding podocyte biology to develop novel kidney therapeutics*,” is the best summary of the aims and the content of this Research Topic. In search for novel therapeutics, the researchers are balancing between the high throughput models with low physiological relevance, such as two-dimensional cultures of immortalized podocytes, and low throughput models with high physiological relevance, including various model organisms. New options in the form of microphysiological systems and kidney organoids may pave the way for identifying new treatment targets and validating drug leads. Concomitantly, revealing new mechanisms, signaling pathways and interactions, accompanied by improved experimental models and novel monitoring techniques, can accelerate the development of podocyte-specific kidney therapies.

## Author Contributions

All authors listed have made a substantial, direct and intellectual contribution to the work, and approved it for publication.

### Conflict of Interest Statement

The authors declare that the research was conducted in the absence of any commercial or financial relationships that could be construed as a potential conflict of interest.

## References

[1] Wiggins RC. The spectrum of podocytopathies: a unifying view of glomerular diseases. Kidney Int. (2007) 71:1205–14. 10.1038/sj.ki.5002222 17410103

[2] Reiser J Sever S. Podocyte biology and pathogenesis of kidney disease. Annu Rev Med. (2013) 64:357–66. 10.1146/annurev-med-050311-163340 23190150 PMC3736800

[3] Lewko B Welsh GI Jankowski MW editors. Podocyte Pathology and Nephropathy. [Internet]. Lausanne: Frontiers Media (2015). 10.3389/978-2-88919-710-1 PMC4585015 26441835

[4] Lin JS Susztak K. Podocytes: the weakest link in diabetic kidney disease? Curr Diab Rep. (2016) 16:45. 10.1007/s11892-016-0735-5 27053072 PMC5064850

[5] Ahlqvist E Storm P Käräjämäki A Martinell M Dorkhan M Carlsson A. Novel subgroups of adult-onset diabetes and their association with outcomes: a data-driven cluster analysis of six variables. Lancet Diab Endocrinol. (2018) 6:361–9. 10.1016/S2213-8587(18)30051-2 29503172

[6] Zhu Q Scherer PE. Immunologic and endocrine functions of adipose tissue: implications for kidney disease. Nature Rev Nephrol. (2018) 14:105–20. 10.1038/nrneph.2017.157 29199276

[7] Musso G Cassader M Cohney S Pinach S Saba F Gambino R. Emerging liver-kidney interactions in nonalcoholic fatty liver disease. Trends Mol Med. (2015) 21:645–62. 10.1016/j.molmed.2015.08.005 26432021

